# Tuberculosis of symphysis pubis in a 17 year old male: a rare case presentation and review of literature

**DOI:** 10.1186/1749-799X-5-63

**Published:** 2010-08-27

**Authors:** Kamal Bali, Vishal Kumar, Sandeep Patel, Aditya K Mootha

**Affiliations:** 1 Deptt of Orthopaedics, PGIMER, Chandigarh, Postgraduate Institute of Medical Education and Research, Sector 12, Chandigarh-160 012, India

## Abstract

Tuberculosis of symphysis pubis is a rare condition with hardly any report of such cases in the last decade. It is necessary to distinguish the entity from more common ones like Osteitis pubis and Osteomyelitis of pubis symphysis by urgent means in order to start the treatment early and thereby minimize morbidity and prevent complications. A rare case of tuberculosis of symphysis pubis in a 17 year old male is described. A high index of suspicion along with an extensive workup including 3-phase bone scan and fine needle aspiration led to the diagnosis. The patient had an excellent outcome following a complete course of multidrug chemotherapy for tuberculosis.

## Background

Inflammation of the symphysis pubis can be non infective (osteitis pubis) or infective(osteomyelitis) in nature. Osteitis pubis is generally a self limiting inflammation of the pubic symphysis secondary to trauma, pelvic surgery, childbirth, or overuse[1]. Osteomyelitis of the pubic symphysis is a rare condition, mostly bacterial in etiology with risk factors being trauma, low grade infection, urological and gynaecological procedures, pelvic malignancies and intravenous drug use[2]. Tuberculosis of the pubis symphysis is still uncommon with 9 cases reported in the past 3 decades. However in the pre-chemotherapy era in the earlier part of the century, upto 100 cases have been reported, which have all been diagnosed in advanced stages. We hereby report a case of tuberculosis of pubic symphysis diagnosed early and treated accordingly with Anti Tubercular Therapy.

## Case presentation

A 17 year old male from low socioeconomic background presented with complaints of a dull aching suprapubic pain for the last 6 weeks. The pain radiated slightly to the left groin. The pain was present continuously throughout the day and it increased on standing and on walking. However coughing, sneezing, voiding or straining at stool did not exacerbate the symptoms. Patient also had a history low grade evening rise in temperature and weight loss of 6 Kg since past 2 months. There was no history suggestive of any trauma, athletic exertion, infection or surgical procedure in the patient. On examination deep tenderness was localized to pubic symphysis. There was no localised swelling and palpation did not reveal any inguinal lymphadenopathy. Rectal examination was also normal.

**Figure 1:**
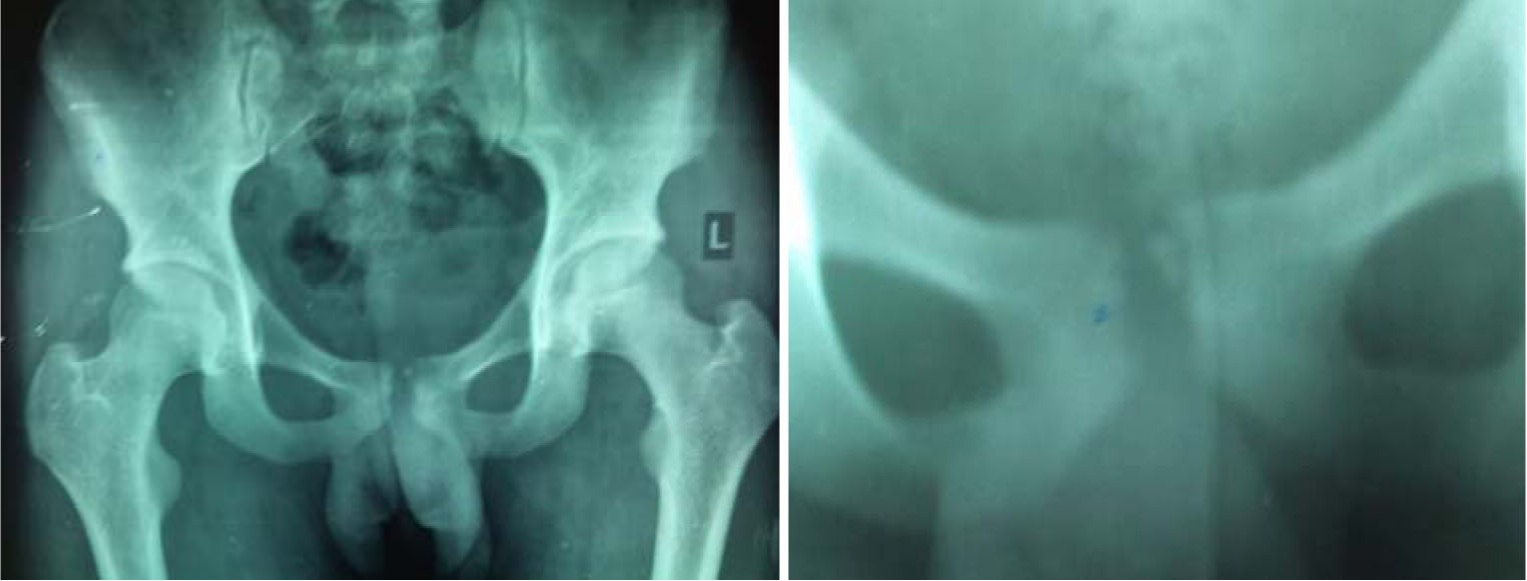
**X ray pictures showing lytic foci in the symphysis pubis**.

Laboratory tests revealed moderately increased white cell counts (15,500/mm^3^), raised Erythrocyte Sedimentation Rate (62 mm/hr) & a positive C Reactive Protein. Mantoux test was nonconclusive. Chest radiographs were normal while the pelvic radiographs revealed rarefaction and lytic changes in bilateral pubis, with more involvement on left side (Fig 1). An initial diagnosis of osteitis pubis was made and the patient started on rest, hot fomentation, NSAIDS and oral ciprofloxacin for 3 weeks.

**Figure 2:**
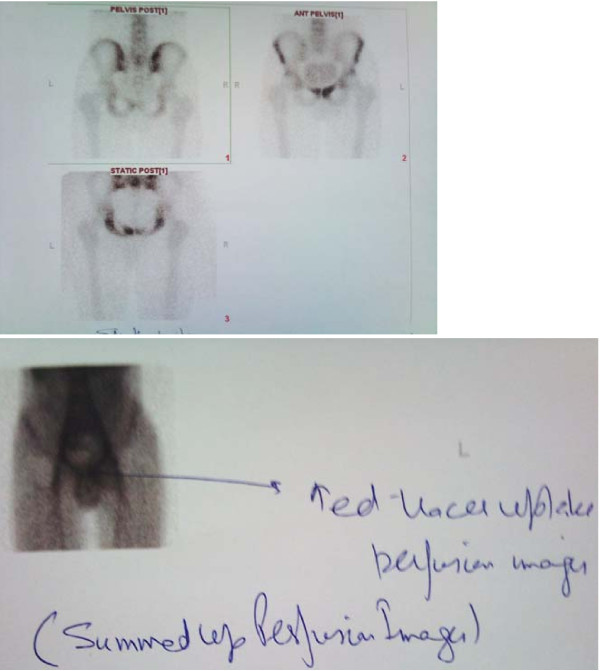
**Technetium 99 m labeled bone scan with increased tracer uptake suggestive of inflammation and infection**.

However the patient did not respond to treatment. A technetium 99 m labeled scan (Fig 2) done at this stage suggested inflammatory (likely infective) pathology of the pubic symphysis. Perfusion and blood pool images showed focal area of increased vascularity in the anterior pelvic region. Delayed anterior, posterior and squatting position static pelvic views showed increased tracer uptake over the superior ramus extending down to the body of left pubic bone and superior ramus of right pubic bone as well. SPECT of pelvic region showed a focus of intense tracer uptake over the superior ramus and body of the left pubic bone and superior ramus of the right pubic bone partially.

**Figure 3:**
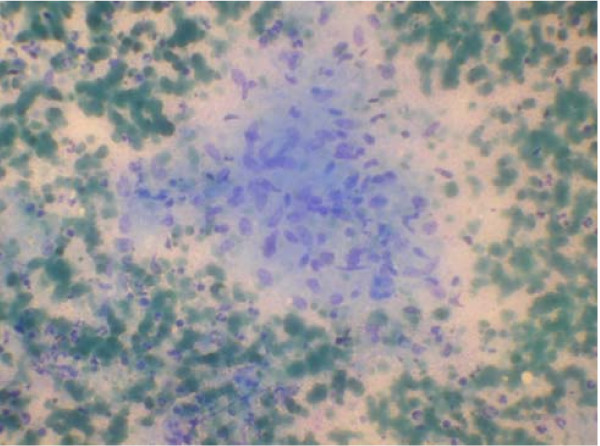
**FNA smear showing epithelioid cell clusters admixed with histiocytes in a background of caseous necrosis and little amount of blood**.

**Figure 4:**
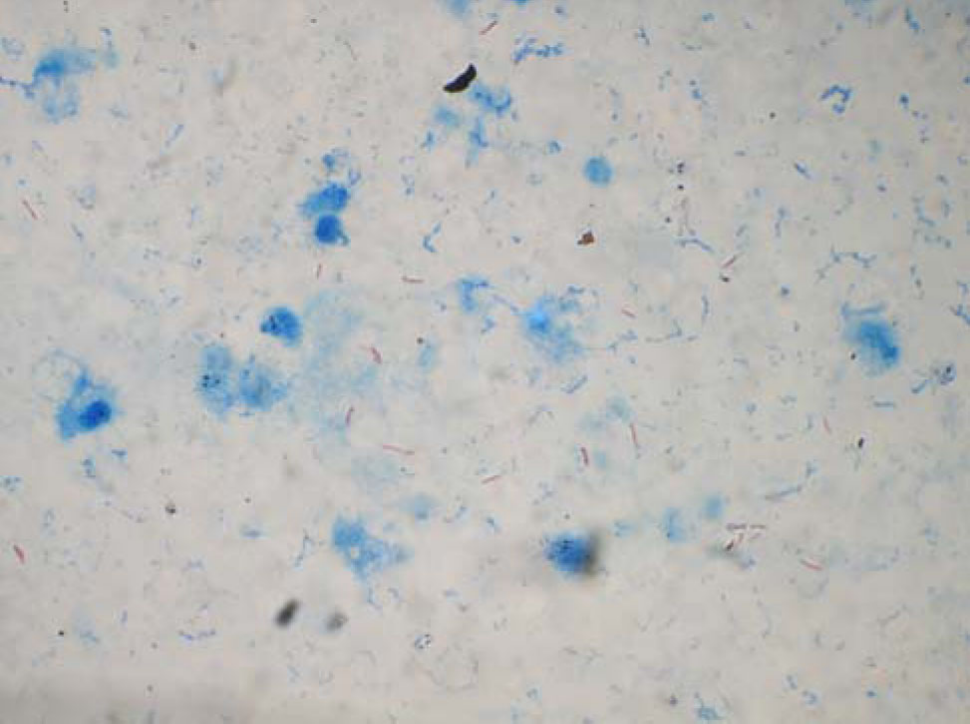
**An AFB stain showing multiple AFB +ve Tuberculous Bacilli**.

MRI of pelvis done also pointed towards infective pathology of the symphysis pubis and further work up showed a positive TB quantiferon test. A fine needle aspiration (FNA) from the pubic symphysis was performed and it showed epithelioid cell clusters admixed with histiocytes in a background of caseous necrosis and little amount of blood ( Fig 3). In context of clinical features and morphological feature on FNA smear, an Acid Fast Bacilli(AFB) stain was performed and it demonstrated multiple AFB positive bacteria (Fig 4).

Once histological evaluation confirmed the diagnosis of tuberculosis, the patient was started on multi drug anti-tubercular chemotherapy comprising of Rifampicin, Isoniazid, Ethambutol and Pyrizinamide. One month following the treatment, patient improved symptomatically and started to gain weight. A repeat radiograph did not show signs of progression. At last follow up after 12 months of chemotherapy, the patient was symptom free with a normal activity level without any signs of recurrence.

## Discussion

Osteoarticular tuberculosis is the second most common form of extrapulmonary tuberculosis next to lymph nodes and constitutes about 13% of all extrapulmonary cases. It is generally accepted that osteoarticular tuberculosis is the result of a haematogenous or lymphatic spread from a reactivated latent focus, usually pulmonary; however, previous infection is not always encountered, and in only 40-50% of the cases, is it possible to demonstrate another active infection site. The commonest site for skeletal tuberculosis is the spine followed by the hip, knee and ankle joints. Tuberculosis can involve literally any bone or joint. With the rising incidence of HIV and multi drug resistant strains, the incidence of extrapulmonary tuberculosis and atypical sites is on rise.

Tuberculosis of the pelvic girdle is primarily limited to the sacroiliac synchondrosis and less frequently with isolated involvement of ilium or ischial tubercle. Symphysis pubis is an unusual site for tubercular infection. Thilesen was the first to describe tuberculosis of symphysis pubis in 1855 followed by Hennies who presented 3 cases in an inaugural address in 1888. The various case series and reviews on the subject are tabulated in Table 1. Some of the largest series are those by Sorell [3] in 1932 (32 cases), Nicholson[3] in 1958 (11 cases), Fares & Pagani [4] in 1966 (27 cases), Dybowski & Makuchowa [5] in 1974 (32 cases). Since the introduction of effective anti-tubercular agents and the general decline in incidence of tuberculosis, involvement of the pubis symphysis appear to have become very rare indeed, if the number of reports indicate the incidence of condition. There are only 9 cases reported in the last 3 decades [6-12].

**Table 1:** Tuberculosis of Symphysis Pubis: Cases reported so far.

YEAR	AUTHOR	NUMBER OF CASES
1888	Hennies	3

1929	Joachimouits	7

1930	Bean HC*	1

1932	Sorell	26

1935	Pytel	1

1938	Gregor	5

1939	Alpert	1

1949	Ficai	2

1951	Clavel	2

1955	Bevan	1

1955	Fairbank	1

1955	Read	1

1958	Nicholson OR	11

1964	Cadili G	1

1966	Fares & Pagani	27

1974	Dybowski & Makuchowa	32

1986	Ker NB	1

1990	Browner U	1

1991	Rozadilla A	1

1992	Mazameque L	1

1995	Tsay MH	1

1997	Benbouazza K	2

2001	Balsarkar DJ	1

2006	Bayrakci K	1

* one case report along with review of 15 cases.

Almost all cases reported have been presented in advanced stages with complications in the form of abscess, sinuses opening to groin or vulva, mass and the morbidity and mortality have been high. Most of the authors have recommended thorough debridement and toileting of the cavities as a treatment strategy. However with the advent of anti-tubercular agents the recovery and prognosis is better. In cases involving complete disruption of symphysis, some form of bridging in the form of plate or bone graft has been advocated[12].

Differential diagnosis in such cases includes osteitis pubis, osteomyelitis, and adolescent osteochondritis of the symphysis pubis. It is essential to differentiate the above entities as the treatment modality for each condition varies. It is even more important to differentiate osteomyelitis and tuberculosis as a delay in diagnosis would result in extensive damage and hence add on to morbidity and residual deformities.

The aetiology of osteitis pubis, or non-infective inflammation of the pubis, is unknown. It is often associated with rheumatic disease, exertion, atheletes, pregnancy, and urological or gynaecological manipulation or surgery [13]. The condition is a self remitting and treatment is conservative in the form of NSAIDS, rest and hot fomentation.

Pyogenic infection of the pubis might be a commoner presentatation than tuberculosis of symphysis pubis. The pathogenesis is usually hematogenic dissemination following trauma, abdominal, urological or gynaecological procedures [2,13].The diagnosis of the condition depends on isolation of the organism. Staph aureus is the most common organism isolated followed by Pseudomonas. Knoeller et al [14] demonstrated that the organism can be cultured even in cases which received antibiotics. Treatment is with appropriate antibiotics while advanced lesions require debridement and toileting.

Clinical presentation however is similar in all the above conditions and includes suprapubic pain sometimes radiating to the groins. Rectus and adductor spasm accounts for the bending noted while standing or walking. Osteitis pubis is self remitting and the symptoms are slightly lighter and decrease with time. Bone scintigraphy and MRI are more sensitive than plain radiographs, especially in the early stages. Three-phase bone scan can be helpful in the differential diagnosis of osteitis and osteomyelitis [15]. Increased uptake in all three phases pleads for osteomyelitis pubis, while increased uptake in the mineralisation or delayed phase only is typical for osteitis pubis. In the very early stages of osteomyelitis pubis, the increased uptake may be limited to one side

## Conclusion

The "key" for the right approach is to exclude the infectious form, osteomyelitis pubis, and tubercular osteomyelitis, and differentiate them by means of aspiration and histological evaluation. Only then can a rational and specific therapy be initiated. In our case, we had a high index of clinical suspicion based on patient profile and initial non response to conservative management. FNAC was diagnostic of Tuberculosis and patient was started on ATT for which he responded. Timely diagnosis and intervention is thus a key to treatment and helped in reducing the morbidity and deformities.

## Consent

Written informed consent was obtained from the patient for publication of this case report and any accompanying images. A copy of the written consent is available for review by the Editor-in-Chief of this journal.

## Competing interests

The authors declare that they have no competing interests.

## Authors' contributions

KB and SP reviewed the literature and wrote the paper. VK and AKM maintained all the records of the patient and followed him. All the authors read and approved the final manuscript.
